# Simulations of Graphene Nanoribbon Field Effect Transistor for the Detection of Propane and Butane Gases: A First Principles Study

**DOI:** 10.3390/nano10010098

**Published:** 2020-01-03

**Authors:** Muhammad Haroon Rashid, Ants Koel, Toomas Rang

**Affiliations:** Thomas Johan Seebeck Department of Electronics, Tallinn University of Technology, Ehitajate tee 5, 12616 Tallinn, Estonia; ants.koel@ttu.ee (A.K.); toomas.rang@ttu.ee (T.R.)

**Keywords:** field effect transistor, graphene nanoribbon, propane, butane, gas sensor, detector, oxygen, humidity, water, nitrogen, carbon dioxide

## Abstract

During the last few years graphene has emerged as a potential candidate for electronics and optoelectronics applications due to its several salient features. Graphene is a smart material that responds to any physical change in its surrounding environment. Graphene has a very low intrinsic electronic noise and it can detect even a single gas molecule in its proximity. This property of graphene makes is a suitable and promising candidate to detect a large variety of organic/inorganic chemicals and gases. Typical solid state gas sensors usually requires high operating temperature and they cannot detect very low concentrations of gases efficiently due to intrinsic noise caused by thermal motion of charge carriers at high temperatures. They also have low resolution and stability issues of their constituent materials (such as electrolytes, electrodes, and sensing material itself) in harsh environments. It accelerates the need of development of robust, highly sensitive and efficient gas sensor with low operating temperature. Graphene and its derivatives could be a prospective replacement of these solid-state sensors due to their better electronic attributes for moderate temperature applications. The presence of extremely low intrinsic noise in graphene makes it highly suitable to detect a very low concentration of organic/inorganic compounds (even a single molecule ca be detected with graphene). In this article, we simulated a novel graphene nanoribbon based field effect transistor (FET) and used it to detect propane and butane gases. These are flammable household/industrial gases that must be detected to avoid serious accidents. The effects of atmospheric oxygen and humidity have also been studied by mixing oxygen and water molecules with desired target gases (propane and butane). The change in source-to-drain current of FET in the proximity of the target gases has been used as a detection signal. Our simulated FET device showed a noticeable change in density of states and IV-characteristics in the presence of target gas molecules. Nanoscale simulations of FET based gas sensor have been done in Quantumwise Atomistix Toolkit (ATK). ATK is a commercially available nanoscale semiconductor device simulator that is used to model a large variety of nanoscale devices. Our proposed device can be converted into a physical device to get a low cost and small sized integrated gas sensor.

## 1. Introduction

Gas sensing has been a critical subject for wide range of applications such as medical, industrial environment, military and aerospace applications. The presence of hazardous and toxic gases may lead to some serious accidents in industrial as well as household environments. There must be some tool to detect the presence of these gases effectively [1,2]. The ultimate goal of gas detection is to obtain high level of sensitivity with high resolution. There presence of very low concentration of desired gases should be detected. However, such high resolution of gas sensor has not been achieved even with solid-state gas sensors [3,4,5]. The main reason of low resolution of these sensors are defects and abrupt fluctuations due to the thermal motion of charge carriers [6], which lead to the creation of noise in these device. Due to this noise, the detection of individual molecules becomes very difficult. Solid-state gas sensors can be categorized into different groups depending on the base of their working principle. The most common categories of such sensors are resistive type sensors, semiconductor gas sensors, impedance type gas sensors (based on alternating current measurements) and electrolyte based gas sensors. The resistive type solid-state gas sensors are the most commonly used gas sensors because their working principle is simple and they have low fabrication cost. The resistance of the constituent semiconductor material changes due to its interaction with target gas. The reason of change in electrical resistance is the transfer of charge carriers between target gas and semiconductor material [7]. In impedance based gas sensors, the frequency response of the device changes in response to the target gas molecules [8]. Whereas, in solid-state electrolyte based gas sensors, the ionic conductivity of the electrolyte changes due to the transportation of holes or electrons from the desired target gas molecules. This change in ionic conductivity is used as a detection signal. Amperometric and potentiometric gas sensors are included in this category [9]. Solid-state gas sensor are very crucial to monitor and control the emission of toxic and hazardous gases. However, they also have some limitations in terms of selectivity, long term stability, sensitivity and reproducibility. The long term stability of electrodes, sensing materials, electrolyte and substrates of solid-state sensors are open challenges. It is also a big challenge to obtain a reliable and accurate reading at high temperatures with such sensors. Due to these reasons, the analysis of desired gases at lower temperature is being done in industries to avoid the issues of inaccuracy and durability of the devices [10].

Although, with an increase in the demand of gas sensors, still there is a need to develop robust, highly sensitive and reversible sensors that work at low temperatures. Gas sensors based on conventional semiconductor materials usually require high operating temperatures. In order to cope with these issues, several efforts are being made to modify the shape and orientations of such materials [11]. Nanomaterials are the promising candidates for the development of gas sensors with low operating temperature and low power consumption. It has been considered that nanomaterials can be used efficiently to detect a large variety of organic/inorganic molecules. The main parameter that dictates the sensitivity of a gas sensing material is its surface-to-volume ratio, which is quite high for nanostructures. This high surface-to-volume ratio allows nanomaterials to adsorb the detectable target molecules effectively and make them suitable candidates to develop efficient gas sensors [12]. In order to overcome the issues found in conventional solid-state gas sensors, graphene has emerged as an exciting and promising candidate to detect a wide range of organic and inorganic materials including gases more efficiently. Extraordinary electronic attributes of graphene and its derivatives make them a promising candidate to replace solid-state gas sensors [13,14,15].

Graphene based gas and inorganic/organic molecule detectors detect the presence of these molecules with different mechanisms. The most popular detection mechanisms are the resistive method, field effect transistors (FET) method and micro-electromechanical system (MEMS) based method. In the resistive method, the change in electrical conductivity in the presence of target molecules is used as a detection signal [16]. In FET based gas detectors, the change in drain-to-source current at some gate voltage is used as a signature to detect the presence of foreign adsorbed particles [17,18]. MEMS based gas sensors have low power consumption, small size, fast response and high sensitivity [19]. In MEMS based sensors, electrical and mechanical components are integrated in the form of a chip. The mechanical component of this sensor converts any physical change in the surrounding into electrical signal [20].

Graphene based field effect transistor (FET) can be a potential candidate to detect the presence of a wide range of chemicals, toxic compounds, biomolecules and gases with better sensitivity compared to that of solid-state sensors. The sensitivity range of these graphene based FETs usually range from parts per billion (ppb) to parts per million (ppm) [21,22,23]. Typically, the gate electrode controls the flow of electric current through FET based gas sensors. Actually, the adsorption of foreign gas molecules and organic compounds effect the concentration of the charge carriers through the graphene layer and consequently the current through the device changes at some gate voltage. Some gas molecules act as donors of charge carriers for graphene and increase the electric current through the device after adsorption. Whereas, some foreign gas molecules act as acceptor for the graphene layer and they reduce the current through the device. This change in electric current is used as a detection signal for gases and other organic/inorganic compounds [24,25]. Even the fluctuation of the conductance can be used as a detection signal [26].

Moreover, propane and butane are the most commonly used fuel for household/industrial environments [27]. These are flammable and toxic gases [28]. In order to avoid fatal explosion accidents, the leakage of these gases in the environment must be detected. Different material processing techniques are used to obtain graphene-based materials like carbon nano-sheets, carbon nanoribbons, and carbon nanotubes with exceptional electronic attributes. Recently, graphene nanoribbons (GNRs) have attracted the interest of researchers due to their distinct electronic properties. GNRs are promising candidate as building blocks of next generation electronics devices [29]. Carbon nanotubes (CNTs) are unzipped with different techniques to get GNRs [30]. Graphene nanoribbon based FETs have very interesting electrical properties that change with the width and direction of the constituent nanoribbons [29,31]. First principles simulations of graphene based FETs to study the doping effects on the IV-characteristics have been reported in the literature [29,32]. Graphene based FET devices and sensors have also been reported in the literature [33,34,35].

In this article, we simulated a novel FET device based on graphene nanoribbons with nanoscale semiconductor device simulator, Quantumwise Atomistix Toolkit (ATK). This simulated device has been used to detect the presence of propane and butane gases. The change in electric current through FET device at different gate voltages in the presence of these gases has been used as a detection mechanism. To the best of our knowledge, this type of device has not been reported in the literature for the detection of propone and butane gases.

## 2. Materials and Methods

The simulations of GNR based FET for the detection of propane and butane gases have been carried out with Quantumwise Atomistix Toolkit (ATK) software package. Graphical user interface of ATK is called Virtual Nano Lab (VNL). ATK-VNL allows atomic scale modeling of nano-systems. This software uses several in-built calculators to solve and calculate transportation properties of quantum systems. Density functional theory (DFT) calculator has been used for the simulations of our proposed device in ATK-VNL. The work flow and mathematical formalism used by ATK-DFT has been given in [36]. All these simulations have been run in a high performance computing environment (HPC) [37]. This HPC has 232 high power computing machines. Each machine has 24 processing units and 48 GB of internal memory. Density of states (DOS) and IV-curves of simulated device in the presence of propane and butane gases have been calculated using eight computing nodes of HPC. With eight computing nodes of HPC, each IV-curve took around more than one week to calculate. A brief description of used materials have been given in the next subsection below. 

### Graphene Armchair and Zigzag Nanoribbons

**Figure 1:**
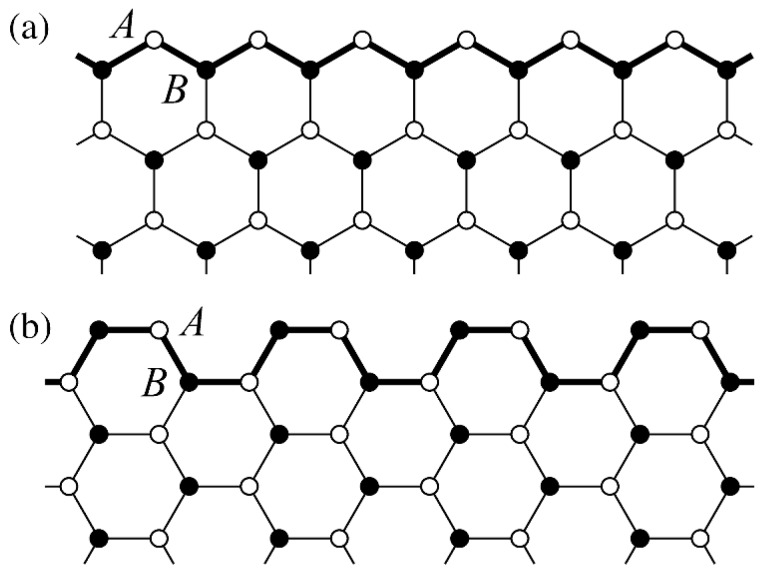
Schematic of (**a**) zigzag graphene nanoribbon; (**b**) armchair graphene nanoribbon reproduced from [38] with permission from SPIE publishers, 2012.

The constituent materials of simulated graphene field effect transistor are armchair graphene nanoribbons (AGNR) and zigzag graphene nanoribbons (ZGNR). The termination pattern of the edge of these structures defines the type of nanoribbon either armchair edge or zigzag edge, as shown in Figure 1 [38]. The structure shown in Figure 1a is zigzag nanoribbon because the termination edge forms a zigzag pattern. Whereas, the structure shown in Figure 1b is armchair nanoribbon because the termination edge forms an armchair pattern. The bandgap of armchair and zigzag nanoribbons change with an increase or decrease in number of carbon atoms in the ribbons [39]. The bandgap of AGNR decreases with an increase in number of carbon atoms in its structure. The bandgap of AGNRs decreases from 3 to 0.75 eV with an increase in number of carbon atoms from 20 to 65 in its ribbon. Whereas, change in bandgap for ZGNR with an increase in number of carbon atoms is different for even and odd number of electrons in its structure. For both cases the bandgap decreases gradually with an increase in number of atoms [39]. GNRs provides perfect interfaces to make junctions at atomic levels. Generally, due to small contact areas, it is very difficult to avoid high contact resistance between metal electrodes and molecular devices. So, this problem could be solved by using metallic GNRs which can be directly connected to the circuits [29]. 

**Figure 2:**
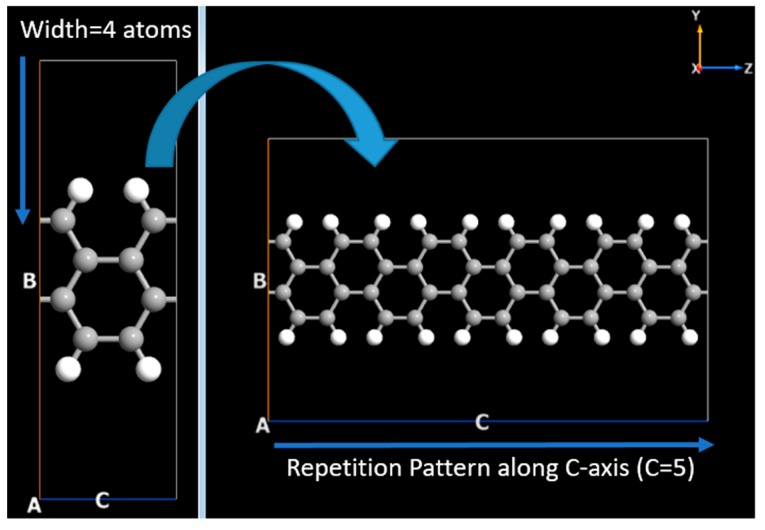
Simulation of armchair graphene nanoribbon for the central region of Field Effect Transistor.

**Figure 3:**
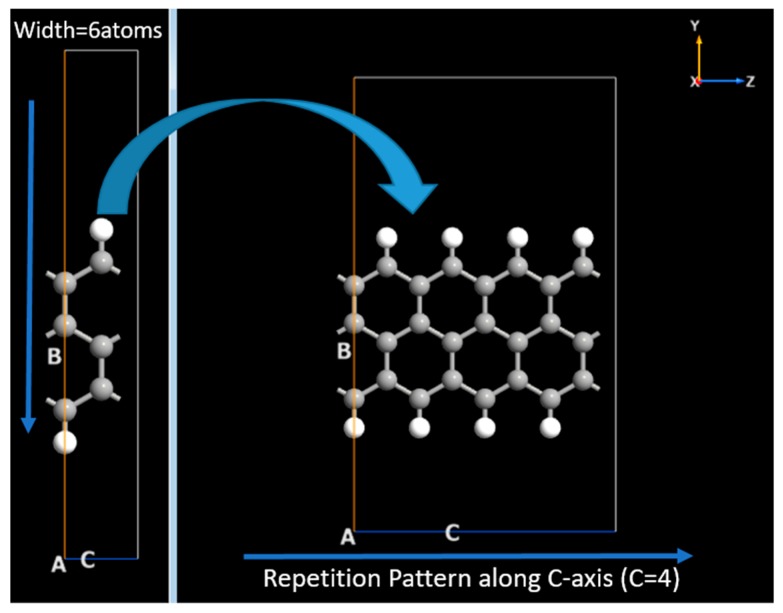
Simulation of zigzag graphene nanoribbons for electrodes of Field Effect Transistor.

Furthermore, the two probe model of GNR based FET has been simulated by using AGNR and ZGNR in ATK-VNL. The builder tool of ATK-VNL is used to simulate FET. All the details have been given in next lines. First of all, the central region of the device have been simulated. The central region of FET consists of AGNR. AGNR with width of four atoms has been created using Nanoribbon Plugin Tool of ATK Software. Afterwards, this GNR has been extended 5 times along C-axis (the repetition pattern) as shown in Figure 2. Whereas A, B and C vectors have been shown in each figure. In the next step, ZGNR has been created with the same plug in tool. However, this time zigzag nanoribbon consists of six atoms, as shown in Figure 3. After creating this ZGNR, its structure has been repeated along C-axis four times and then a copy of this structure has been made. These two structures will be used to form electrodes of FET by connecting them to the central region of the device.

**Figure 4:**
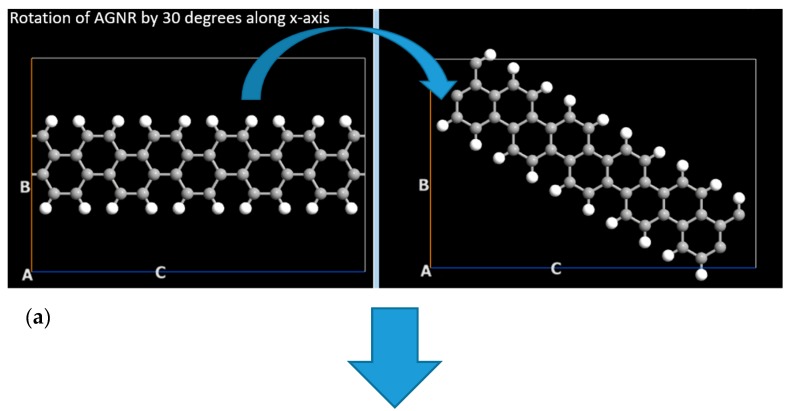

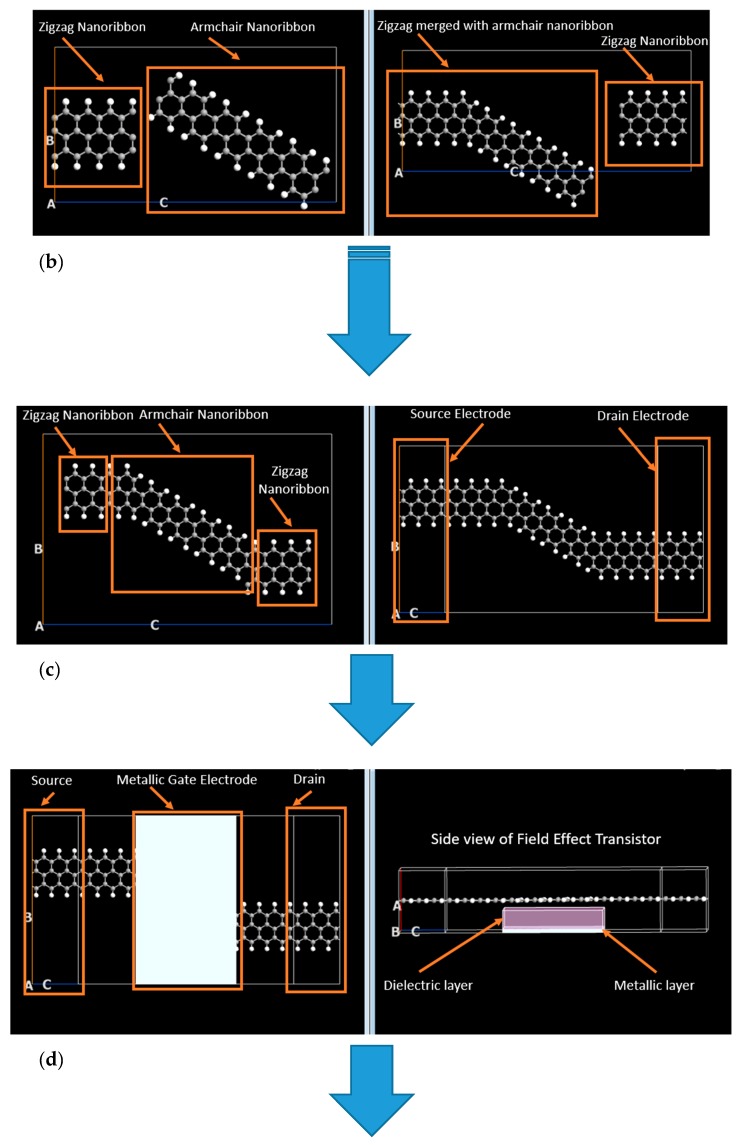

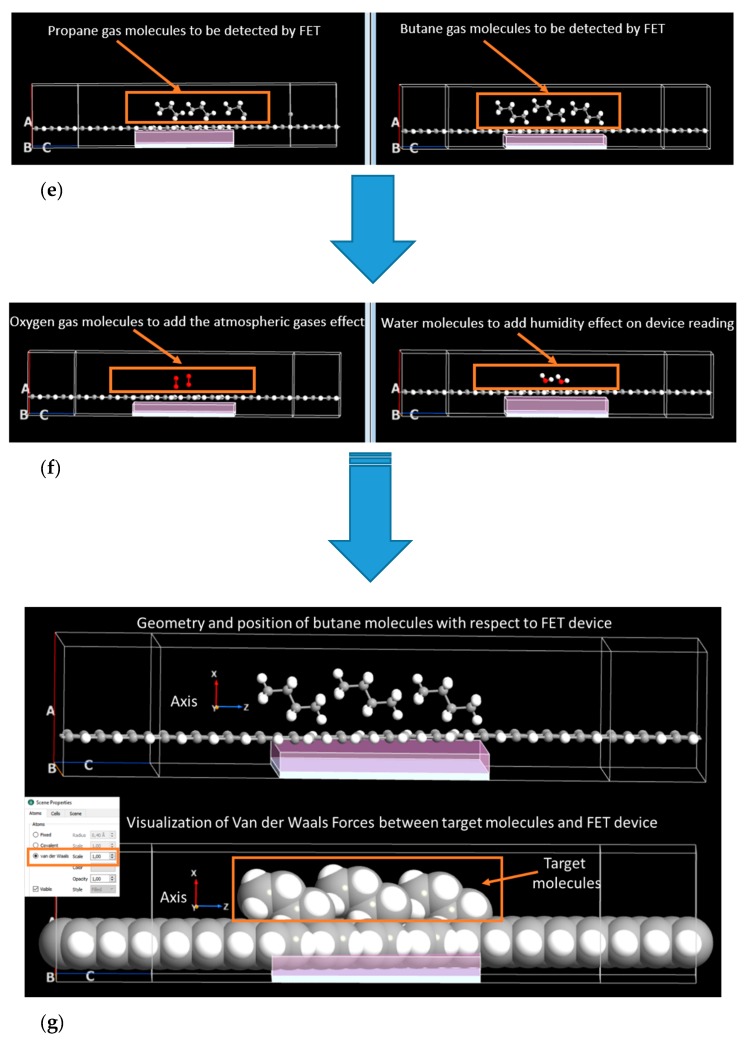
Simulation steps for graphene zigzag and armchair nanoribbon based field effect transistor for the detection of propane and butane gases under the influence of atmospheric oxygen and humidity.

After that, the next step is to join the central region (Figure 2) with the electrodes (Figure 3) to form a z-shaped structure. For this purpose, armchair graphene nanoribbon has been rotated by 30 degrees along X-axis (the axis have been shown in the upright position of Figure 2 and Figure 3, as shown in Figure 4a). Now, AGNR is ready to be joined with ZGNR (electrodes). The next step is to merge ZGNR with AGNR to form a z-shaped structure, as shown in Figure 4b. After merging these cells, we get a z-shaped structure in which AGNR is in the center (central region) whereas ZGNRs are on the left and right side of this structures. These ZGNRs form the source and drain electrodes of FET, as shown in Figure 4c. In the next step, dielectric material and gate electrode have been deposited on this structure to get FET device, as shown in Figure 4d. The permittivity of the dielectric material has been chosen as 4*ε*_o_ and a very thin metallic layer has been deposited on it to form a gate electrode of field effect transistor. The lengths of source and drain electrodes are approximately 7 Å. In next step, three molecules of propane, three molecules of butane and both propane & butane (four molecules of butane and one molecule of propane) have been exposed to the FET device in three different experiments, as shown in Figure 4e. In order to add the influence of atmospheric gases and humidity, oxygen and water molecules have been mixed with the desired target gases (propane and butane). Two oxygen molecules and two water molecules have been exposed to the device, as shown in Figure 4f. In the simulated device, these molecules have also been mixed with propane and butane target gases in two different experiments. 

Furthermore, an in-built Merger Tool of ATK-VNL has been used to expose the target molecules (i.e., propane, butane, oxygen, water molecules) to the simulated FET device (Figure 4d). ATK-VNL has an inbuilt Move Tool that is used to move (in *X*, *Y*, *Z* coordinates) the target molecules with respect to the simulated FET device. The position and geometry of each butane molecule along with *X, Y, Z* axis have been shown as an example in Figure 4g. It can be seen in this figure that the geometry of all butane molecules are almost identical to each other with respect to the FET device. Similar atoms (oxygen atoms in case of butane molecules) of all target molecules are facing to the FET device. The same procedure has been adopted for exposing other target molecules to the simulated FET device. All target molecules have been kept at an optimal distance of few Angstroms to the FET device, ensuring that Van der Waals Forces are acting between target molecules and FET device. Graphical user interface of ATK-VNL software has been used to confirm that Van der Waals Forces are acting between target molecules and FET device, as shown in Figure 4g. A more comprehensive detail about simulating graphene nanoribbon based FET in ATK-VNL can be found at this reference [40]. The reason of choosing this small number of gas molecules in these simulations is to reduce the computation time in HPC environment. The change in density of states (DOS) and IV-characteristics of FET have been calculated in the presence of propane and butane as target molecules. 

## 3. Results and Discussion

In this section, the results obtained from simulations have been presented and discussed in detail. The DOS and IV-characteristics of simulated FET based sensor have been calculated. 

### 3.1. Density of States of Simulated Graphene Nanoribbon Field Effect Transistor Device

**Figure 5:**
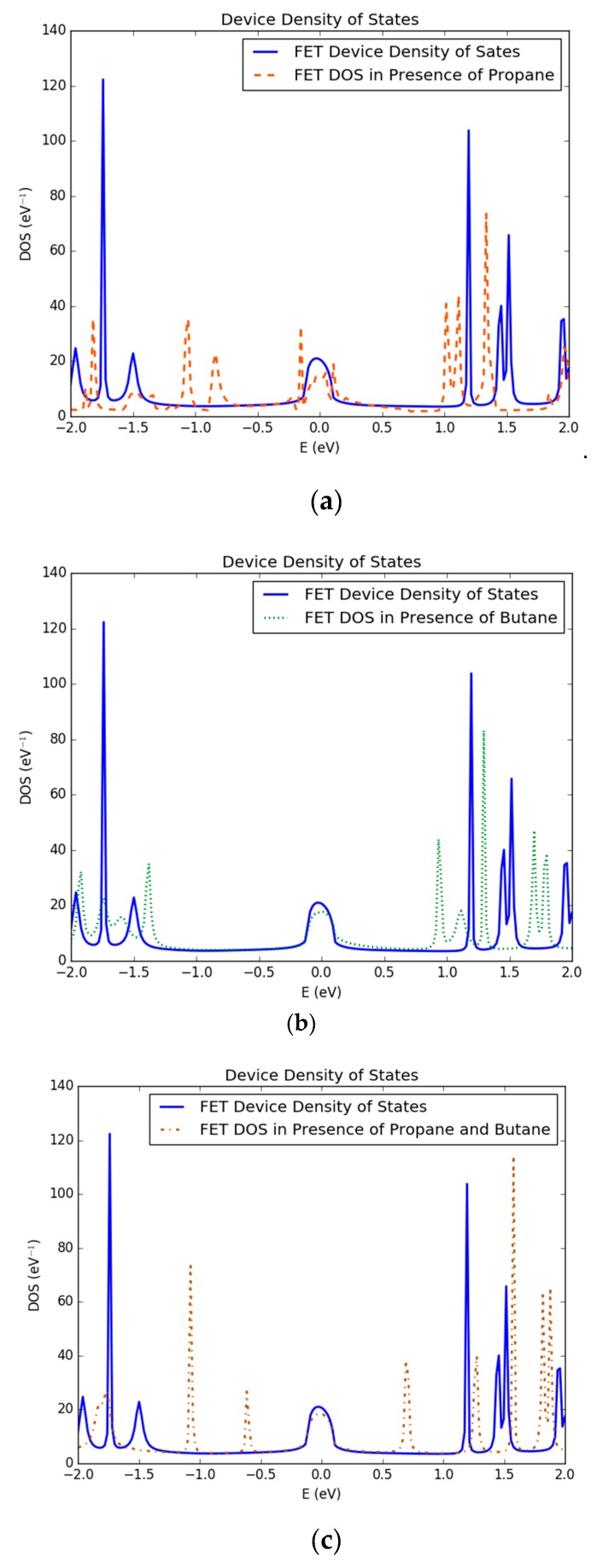
Change in density of states (DOS) of simulated filed effect transistor in presence of (**a**) propane gas molecules; (**b**) butane gas molecules; (**c**) both propane and butane gas molecules.

A significant and distinct change in the DOS of FET device have been observed in the presence of different target gas molecules. A comparison of DOS of simulated FET device in the absence of any target gas molecules and in the presence of three propane gas molecules has been shown in Figure 5a. It can observed that many new energy states have been introduced by propane gas molecules both above and below the fermi level of the device compared to that of the reference simulated device (without any target gas), as shown in Figure 5a. Many new energy spikes can be observed at energy levels of −1.8, −1.2, −0.8, 1.0 and 1.3 eV in FET device in the presence of propane gas.

Furthermore, the presence of three butane gas molecules affected DOS of FET differently compared to that of propane gas molecules, as shown in Figure 5b. Many new energy states can be observed at energy levels of −1.9, −1.4, 0.9, 1.6 and 1.8 eV, approximately. These energy states were not present in the presence of propane molecules. Similarly, a distinct change in DOS can be observed in the device when it is exposed to both propane and butane gas molecules simultaneously, as shown in Figure 5c. It can be observed that the presence of both gases introduced new energy states in FET device. New energy spiked can be observed at energy levels of −1.1, −0.6, 0.6, 1.3, 1.6, 1.8 and 1.9 eV approximately, as shown in Figure 5c.

### 3.2. Current-Voltage Characteristics of Simulated Graphene Nanoribbon Field Effect Transistor Device in Presence of Only Propane and Butane Molecules

**Figure 6:**
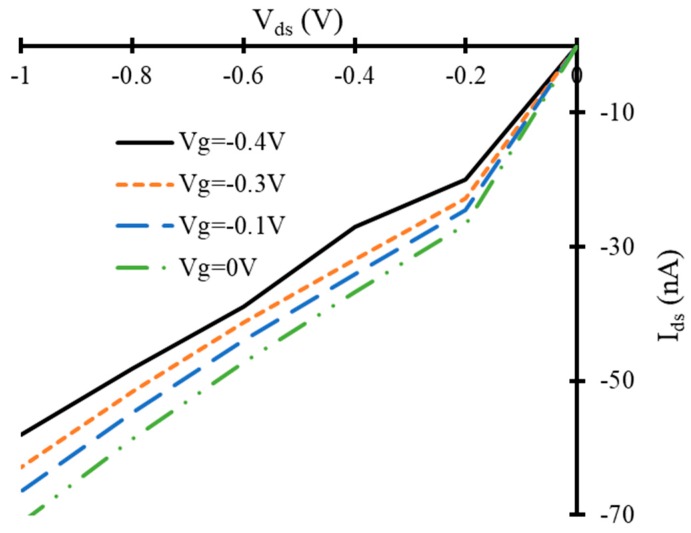
*V_ds_* versus *I_ds_* curves of simulated field effect transistor at different gate voltages in the absence of target gas molecules.

Drain to source current (*I_ds_*) and voltage (*V_ds_*) curves of simulated FET have been calculated for different applied gate voltages. The simulated device (in absence of any target gas molecules) showed depletion mode MOSFET like behavior. A decrease in *I_ds_* versus *V_ds_* has been observed with an increase in negative gate to source voltage (*V_gs_*), as shown in Figure 6. The same IV-curves for the gate voltages of −0.1, −0.3 and −0.5 V have been calculated in the presence of three propane molecules, three butane molecules and both gases (four molecules of butane and one molecule of propane) in three different experiments. In the presence of target gas molecules, a significant change in IV-curves of FET device has been observed for the same voltage biased conditions compared to that of the device in the absence of target gas molecules. This change in IV-characteristics can be used as a detection signal to detect the presence of propane and butane gases.

In our previously published work [31], we used purely resistive method based on pristine AGNR to detect propane and butane gases. However, in this article, we used FET based device in which the electric current not merely depends on applied bias voltage (*V_ds_*), but also on gate voltage. The adsorbed target gas molecules act as donors or acceptors of charge carriers for the graphene. If they act as donors, they change (increase) the concentration of charge carriers in graphene. Consequently, the electric current through the graphene based device increases in the presence of adsorbed target molecules under same bias condition. When the foreign target molecules behave like acceptors of charge carriers, they decrease the current through the graphene based device [41]. In resistive methods of graphene based gas detectors, it is comparatively easy to realize this change in conductivity of graphene. Because in such type of devices, graphene behaves like a resistive strip which is only a function of external applied voltage. Nevertheless, in FET based graphene sensors, the change in charge carriers through the graphene channel is also a function of gate voltage and hence could be more accurate. So one has to keep in mind the effect of gate voltage as well as *V_ds_* on the IV-characteristics of FET based sensor.

**Figure 7:**
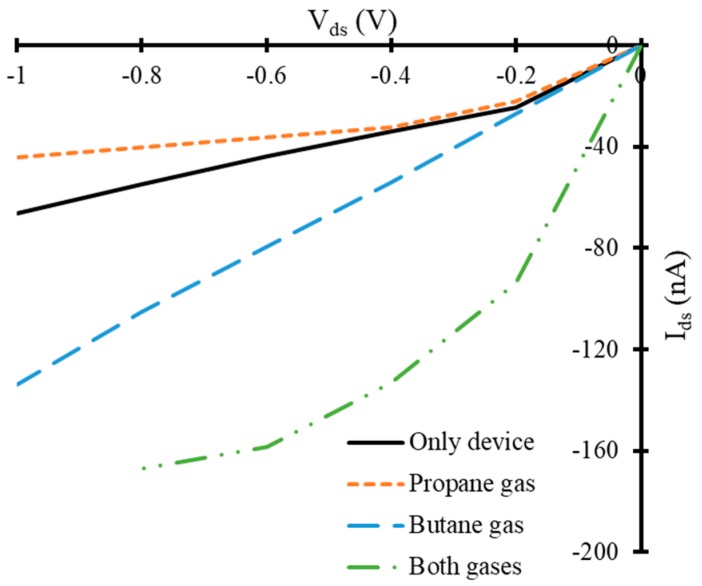
*V_ds_* versus *I_ds_* curves of simulated field effect transistor at −0.1 V gate voltage in the presence of target gas molecules.

Furthermore, our simulated device exhibited a considerable change in IV-characteristics in the presence of the target gases. The reference FET device showed a current range between −24 nA to −66 nA at *V_gate_* = −0.1 V and at a fixed *V_ds_*, shown with a solid black line in Figure 7. A decrease in *I_ds_* has been observed in the presence of propane as a target gas, at *V_gate_* = −0.1 V, as shown with orange dotted line in Figure 7. The current range of FET based device in the presence of propane target is between −22 nA to −44 nA, which is less than that of reference device. It seems that propane molecules may have acted like acceptors of charge carriers for graphene and reduced the charge carrier concentration in the device. Consequently, a decrease in current has been observed in the presence of propane molecules. The device showed a sufficient increase in *I_ds_* in presence of butane gas molecules compared to that of reference device. The range of the *I_ds_* is between −26 nA to −134 nA approximately in the presence of butane gas, shown with blue color dashed line in Figure 7. It seems that butane molecules may have acted like donors of charge carriers for graphene and increased the charge carrier concentration in the device. Consequently, an increase in current has been observed in the presence of butane molecules. A dramatic increase in *I_ds_* has been observed when FET device is exposed to four butane and one propane gas molecules simultaneously. The range of *I_ds_* in this case is between −93 nA to −167 nA at *V_gate_* = −0.1 V, which is quite high compared to that of the reference device. This high amount of current through the device may be due to the donor like dominated effect of four butane molecules compared to that of one propane molecule (acceptor like behavior), which are exposed to the device simultaneously. This curve has been shown with a dotted-dashed green colored line in Figure 7.

**Figure 8:**
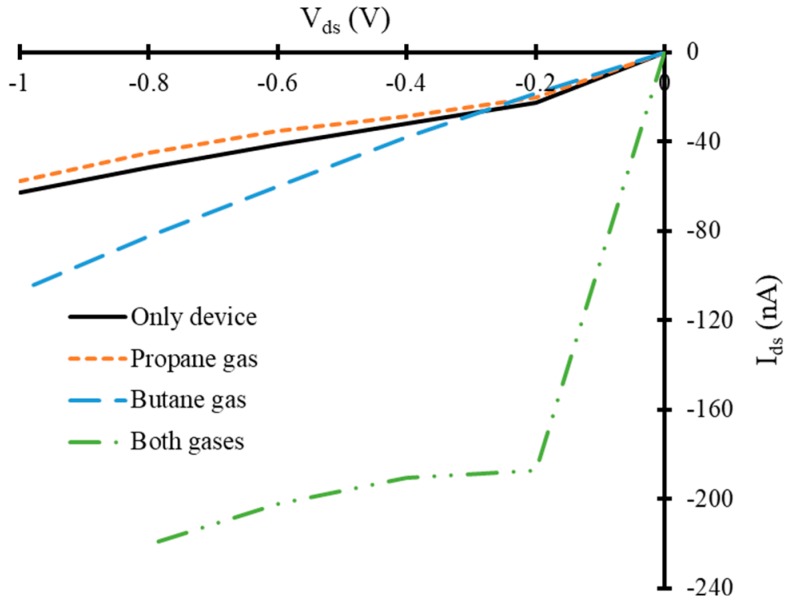
*V_ds_* versus *I_ds_* curves of simulated field effect transistor at −0.3 V gate voltage in the presence of target gas molecules.

**Figure 9:**
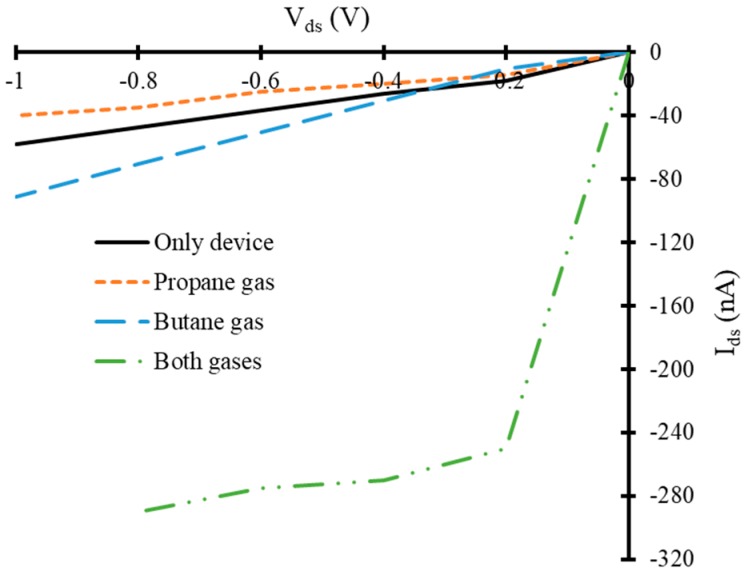
*V_ds_* versus *I_ds_* curves of simulated field effect transistor at −0.5 V gate voltage in the presence of target gas molecules.

However, an increase in negative bias gate voltage reduced the overall *I_ds_* through reference FET, as it is a depletion mode n-channel FET. A similar trend of increase and decrease in *I_ds_* at *V_gate_* = −0.3 V for the same *V_ds_* has been observed with respect to the reference FET device, as shown in Figure 8. In presence of propane gas molecules a decrease in *I_ds_* at this *V_gate_* has been observed compared to that of reference FET. The range of *I_ds_* in this case is between −20 nA to −57 nA, approximately. Whereas butane gas increased the *I_ds_* compared to that of reference FET and the range of current is between −18 nA to −106 nA. The simultaneous presence of both gases increased the *I_ds_* in similar manner that is observed in previous cases. The range of electric current under this bias condition is between −187 nA to −220 nA, which is quite high compared to that of the device at *V_gate_* = −0.1 V, as shown in Figure 8. Finally, the IV-characteristics of the device have been calculated at *V_gate_* = −0.5 V, as shown in Figure 9. It also exhibited a similar trend but with different current ranges compared to that of the previously discussed cases.

### 3.3. Influence of Oxygen and Water Molecules on the Current-voltage Characteristics of Simulated Graphene Nanoribbon Field Effect Transistor

**Figure 10:**
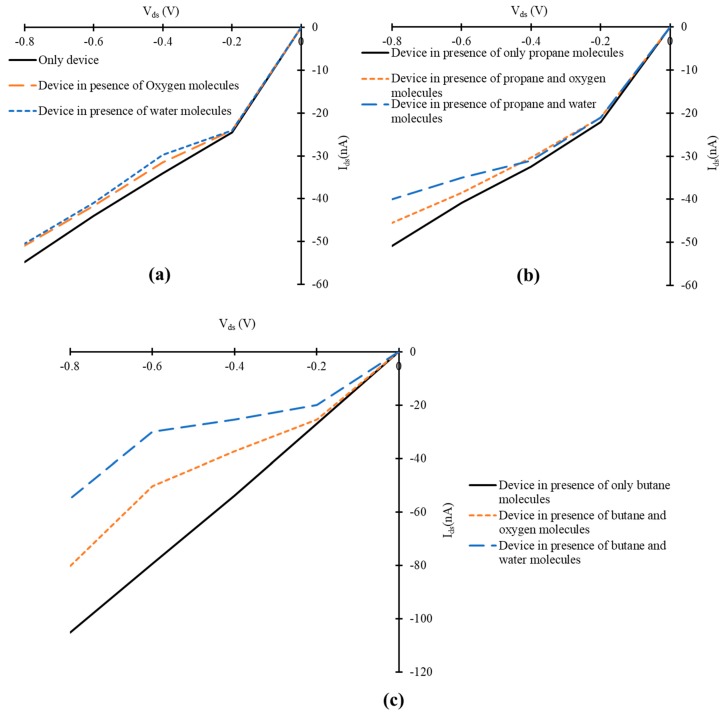
*V_ds_* versus *I_ds_* curves of simulated field effect transistor at −0.1 V gate voltage in the presence of (**a**) only water and oxygen molecules; (**b**) propane, water and oxygen molecules; (**c**) butane, water and oxygen molecules.

In Section 3.2. the simulated graphene based FET device is exposed to only propane and butane molecules in a controlled environment, where the influence of the atmospheric species like oxygen, carbon dioxide, water vapors and humidity have been neglected. The purpose of the work presented in this article is to access the feasibility of the simulated device for the detection of propane and butane gases. But for real-time application of such device, the influence of other atmospheric parameters cannot be ignored. Therefore, we also studied the effect of oxygen and water molecules on IV-characteristics of simulated structure. For this purpose, additional simulations have been done. In two different experiments, the simulated FET has been exposed to only two oxygen molecules and only two water molecules. In third and fourth experiment, three propane molecules have been detected in the presence of two water molecules and two oxygen molecules individually. In fifth and sixth experiment, three butane molecules have been detected in the presence of two water molecules and two oxygen molecules individually. The IV-curves of simulated device at *V_gate_* = −0.1 and fixed *V_ds_* have been calculated. A slight decrease in *I_ds_* of the simulated FET has been observed in the presence of two oxygen molecules, as shown in Figure 10a. At exposure to two water molecules, the FET device also exhibited a decrease in source-drain current, as shown in Figure 10a. In case of exposure to two water molecules, the decrease in current of device is more compared to that of the oxygen molecules. Similarly, a decrease in *I_ds_* has been observed, when water and oxygen molecules are mixed with propane gas molecules, as shown in Figure 10b. At the exposure of the device to butane molecules mixed with water molecules simultaneously, the device showed a decrease in *I_ds_*, as shown in Figure 10c. Whereas, a similar effect on *I_ds_* has been observed in the presence of mixture of butane and oxygen molecules, as shown in Figure 10c.

The presence of both oxygen and water molecules degraded the device performance in terms of reduction of detectable current for propane and butane gases. A careful calibration of such physical sensor is required to nullify the environmental effects on the actual reading of the device in the presence of desired target molecules. The presence of oxygen content in the atmosphere has a strong influence on the electrical properties of graphene. The oxygen content of the air strongly affects the electrical resistance of graphene which depends on the exposure time. The electrical conductance of graphene reduces with passage of time at its exposure to the oxygen content of the air. Therefore, the change in device performance may be expected as exposure time increases [42]. Similarly, the presence of water content in the air also has an effect on the electrical properties of the graphene as water molecules act as p-type dopant for graphene and change its electronic properties. Therefore, the device should be encapsulated for stable operation [43,44,45].

Moreover, in ambient atmospheric conditions, the effect of nitrogen (N_2_) and carbon dioxide (CO_2_) on the electrical conduction properties of graphene based sensors are negligible. Whereas, water molecules have strong influence on the conductivity of graphene. The considerable response of graphene to the water molecules makes it highly suitable for humidity sensor applications [46]. Therefore, N_2_ and CO_2_ gases have not been considered in these simulations. The purpose of these simulations is to investigate the feasibility of simulated FET device for the detection of solely propane and butane molecules for household and industrial environments. However, the concentration of methane (CH_4_) gas is increasing day by day due to green house effect. Several efforts have been done for the development of CH_4_ gas sensors [47,48,49,50,51,52,53,54,55,56,57,58]. The detection of CH_4_ with our proposed device for climatic and household applications can be considered as future work. These simulation results are convincing in terms of the ranges of detectable electric current values. In our previous work [31], the differentiation between electric current values in the presence of the these target gases was difficult for each individual gas and the combination of both gases. In current work, the behavior of FET device and current values are more obvious. However, the trend of increase or decrease of current values in IV-curves for both devices shows a conflict. The possible reason of this conflict could be the involvement of the effect of gate voltage and addition of zigzag nanoribbons as electrodes for the FET device that was absent in our previously simulated device [31]. The physical fabrication of this type of device could be challenging due to the handling and processing of nanoribbons at atomic level. In near future, as the graphene technology will evolve, the physical fabrication of such type of devices will be possible.

## 4. Conclusions

The goal of the work that has been presented in this article is to access the potential of graphene nanoribbon based FET for the detection of propane and butane gases. Armchair graphene nanoribbons and zigzag nanoribbon have been used to develop FET device. In the absence of any target gas molecules, the IV-characteristics of the simulated device are similar to n-channel depletion mode FET. In the proximity of target gas molecules, a change in source-to-drain current of FET at different gate voltages has been observed. This change is distinct for each specific target gas i.e., propane and butane. Our simulated FET device also exhibited a noticeable change in the density of states in the proximately of these target gases. The change in source-to-drain current of FET in the presence of target gas molecules has been used as a detection signal for the leakage detection of these gases. The influence of atmospheric factors like the presence of water and oxygen molecules on the proposed device have also been investigated. It has been observed that the presence of these molecules also affected the IV-characteristics of the device. A careful calibration of such physically fabricated device is required to nullify the effect of atmospheric factors and get the correct reading for desired target gases. Our proposed device could be a promising candidate to replace conventional solid-state gas sensors due to its exceptional electronic properties and compact size. Theoretically, it is possible to simulate such type of FET sensors. However, the physical fabrication of these kind of device could be a challenge due to extremely small dimensions of graphene nanoribbons that make it difficult to handle at atomic levels. Moreover, during physical fabrication of our proposed FET, there is a need of some suitable substrate like SiC or Si for the deposition of graphene. Our nearest future intension is to develop graphene based FET device for the detection of propane and butane gases.
